# Biosynthetic gene cluster synteny: Orthologous polyketide synthases in *Hypogymnia physodes, Hypogymnia tubulosa*, and *Parmelia sulcata*

**DOI:** 10.1002/mbo3.1386

**Published:** 2023-10-17

**Authors:** Nadim Ahmad, Manfred Ritz, Anjuli Calchera, Jürgen Otte, Imke Schmitt, Thomas Brueck, Norbert Mehlmer

**Affiliations:** ^1^ Department of Chemistry, Werner Siemens Chair of Synthetic Biotechnology, TUM School of Natural Sciences Technical University of Munich (TUM) Garching Germany; ^2^ Senckenberg Biodiversity and Climate Research Centre (SBiK‐F) Frankfurt am Main Germany; ^3^ Institute of Ecology, Evolution and Diversity Goethe University Frankfurt Frankfurt am Main Germany

**Keywords:** biosynthetic gene cluster, long‐read sequencing, *Parmelia sulcata*, Parmeliaceae, phylogeny, polyketide synthesis

## Abstract

Lichens are symbiotic associations consisting of a photobiont (algae or cyanobacteria) and a mycobiont (fungus), which together generate a variety of unique secondary metabolites. To access this biosynthetic potential for biotechnological applications, deeper insights into the biosynthetic pathways and corresponding gene clusters are necessary. Here, we provide a comparative view of the biosynthetic gene clusters of three lichen mycobionts derived from *Hypogymnia physodes, Hypogymnia tubulosa*, and *Parmelia sulcata*. In addition, we present a high‐quality PacBio metagenome of *Parmelia sulcata*, from which we extracted the mycobiont bin containing 214 biosynthetic gene clusters. Most biosynthetic gene clusters in these genomes were associated with T1PKSs, followed by NRPSs and terpenes. This study focused on biosynthetic gene clusters related to polyketide synthesis. Based on ketosynthase homology, we identified nine highly syntenic clusters present in all three species. Among the four clusters belonging to nonreducing PKSs, two are putatively linked to lichen substances derived from orsellinic acid (orcinol depsides and depsidones, e.g., lecanoric acid, physodic acid, lobaric acid), one to compounds derived from methylated forms of orsellinic acid (beta orcinol depsides, e.g., atranorin), and one to melanins. Five clusters with orthologs in all three species are linked to reducing PKSs. Our study contributes to sorting and dereplicating the vast PKS diversity found in lichenized fungi. High‐quality sequences of biosynthetic gene clusters of these three common species provide a foundation for further exploration into biotechnological applications and the molecular evolution of lichen substances.

## Graphical abstract

The new *Parmelia sulcata* genome from the metagenomic sample was evaluated with *Hypogymnia physodes* and *Hypogymnia tubulosa* to identify orthologous polyketide synthase (PKS)‐related biosynthetic gene clusters (BGC). The phylogenetic tree highlights the relation of investigated sequences. Evaluation of orthologous PKS‐related BGCs emphasizes the high homology of biosynthetic core genes.

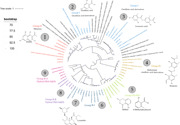

## 1 INTRODUCTION

In the past, lichens were considered a mutualistic symbiosis consisting of a fungus and partners capable of photosynthesis, such as algae or cyanobacteria (Plitt, 1919). Recent research has indicated that lichen individuals may contain not only the primary fungus responsible for lichen formation (mycobiont) and the primary photosynthetic partner (photobiont) but also additional fungi from other phyla and other organisms. Among these are Basidiomycota (Spribille et al., 2016) or Ascomycota (Muggia & Grube, 2018), as well as various types of bacteria and other algae (Aschenbrenner et al., 2016; Smith et al., 2020). Today, lichens are often referred to as ecosystems (Hawksworth & Grube, 2020) or holobionts (Rolshausen et al., 2022). Over the past few decades, the distinct secondary metabolite profile of these composite organisms has attracted growing interest (Ranković & Kosanić, 2019). A plethora of natural products are synthesized by lichen mycobionts (Huneck & Yoshimura, 1996). Among the latter, the most prominent classes include depsides, depsidones, dibenzofurans, and phenolic compounds (Elix & Stocker‐Wörgötter, 2008; Calchera et al., 2019). These compounds exhibit bioactivities of antimicrobial (Kosanić et al., 2013; Ranković & Kosanić, 2019; Ristić et al., 2016; Sisodia et al., 2013), antifungal (Karabulut and Ozturk, 2015), anti‐inflammatory (Joshi et al., 2019), antioxidant (Goga et al., 2020; Kosanić & Ranković, 2019), and antitumoral (Kosanić et al., 2013; Solárová et al., 2020) nature. Examples of medically relevant compounds include gyrophoric acid, atranorin, and physodic acid (Cardile et al., 2017). Compounds like physodic acid, evernic acid, atranorin, and usnic acid displayed an inhibitory effect on metabolic enzymes (Boustie & Grube, 2005; Calchera et al., 2019).

To utilize these compounds biotechnologically, it is essential to have a thorough understanding of their biosynthesis. The compounds mentioned above are classified as polyketides and are created through multiple catalytic cycles in which elongation units are linked to a starter molecule by mega‐enzyme polyketide synthases (PKS) (Crawford & Townsend, 2010). In the context of fungal PKS, reducing PKS (R‐PKS) and nonreducing PKS (NR‐PKS) are distinguished based on their domain composition and the extent of reductive processing involved in their catalytic cycles. R‐PKSs contain additional domains responsible for reducing the intermediate polyketide chain during synthesis, while NR‐PKSs lack these domains and thus do not perform any reduction steps (Cox, 2023). Conserved structures in R‐PKS from N‐ to C‐termini consist of ketosynthase (KS); acyl transferase (AT); dehydratase (DH); C‐methyl transferase (cMT); enoylreductase (ER); ketoreductase (KR); and acyl carrier protein (ACP) domains (Cox, 2023; Dutta et al., 2014). In contrast, unique PKS domains comprising starter unit:acyl‐carrier protein transferases (SAT) and a product template (PT) domain are observed in NR‐PKS (Crawford & Townsend, 2010; Huitt‐Roehl et al., 2015). SAT domains link the chain‐initiating compound to the enzyme, while the PT domain is responsible for regulating the cyclization reactions that convert highly reactive, fully elongated intermediates into specific aromatic compounds (Crawford & Townsend, 2010; Crawford et al., 2009; Huitt‐Roehl et al., 2015; Li et al., 2010). NR‐PKS domains are mostly organized in the following order SAT‐KS‐AT‐PT‐ACP‐ACP‐TE (Wang et al., 2021). Other occurring units comprise AMP‐binding sites (A), carrier proteins (CP), and the terminal domain (TD); these are however dedicated to hybrid PKS‐NRPS (Meier & Burkart, 2011; Boettger & Hertweck, 2013; Calchera et al., 2019).

Next‐generation sequencing (NGS) has enabled the identification of potentially involved genes in natural product formation through deep genome sequencing. If multiple genes that are involved in the formation of a specific secondary metabolite are coregulated and located in proximity (not dispersed throughout the genome), they are referred to as a biosynthetic gene cluster (BGC) (Keller, 2019; Keller et al., 2005; Medema et al., 2015; Pizarro et al., 2020; Rokas et al., 2018). Recent studies have revealed that BGCs located within lichenized fungi are responsible for the production of various secondary metabolites, such as pigments, terpenes, and polyketides (Kealey et al., 2021; Kim et al., 2021; Llewellyn et al., 2023; Singh, 2023; Singh et al., 2021b).

To identify particular BGCs, it is essential to gain access to the genomic composition of the symbiotic partners. This can be accomplished by sequencing the entire meta‐genome, which provides a comprehensive view of all BGCs present in the organisms involved in the lichen symbiosis. This approach may facilitate uncovering interactions between the multiple species involved in the symbiosis at the level of BGCs (Aschenbrenner et al., 2016). Therefore, metagenomic tools are deployed to divide the obtained metagenomes taxonomically into respective bins. This renders symbiotic partners, which are challenging to cultivate, accessible to genetic interrogation (Muggia et al., 2010). The genome of the mycobiont often harbors most of these BGCs (Pizarro et al., 2020), which are activated in response to environmental stimuli (Chiang et al., 2011; Zheng et al., 2020). In addition, various structurally similar compounds may be encoded by one BGC (Martinet et al., 2019; Singh et al., 2022; Wasil et al., 2013).

Short‐read sequencing methods can have limitations in accurately representing isoforms among different species in a sample. This is because short reads may not span across complex genomic or repetitive regions, as well as capture the full extent of genomic diversity in mixed microbial communities. As a result, it can be challenging to confidently assemble the complete genomes of all species present in the sample (Bickhart et al., 2022; Cuscó et al., 2021; Tsai et al., 2016). To overcome these limitations, long‐read sequencing techniques are utilized instead. These technologies have the potential to generate more contiguous genome assemblies and improve our ability to accurately identify BGCs and other functional genetic elements in complex microbial communities, resulting in high‐quality data output (Xie et al., 2020). Furthermore, nucleotide variances among symbionts can be detected by long reads (Chen et al., 2022).

In this study, we present a new lichen metagenome of *Parmelia sulcata* (PSU) (BioSample SAMN35345252) and the BGCs of the mycobiont. *P. sulcata* is one of the most common species of lichen‐forming fungi worldwide. It is widely distributed in temperate and cold regions of both hemispheres and typically grows on the bark of trees. The lobed thallus has a light gray surface, usually with white ridges and soredia*. P. sulcata* belongs to a species complex, characterized by high genetic diversity (Crespo et al., 1997, 1999; Feuerer & Thell, 2002; Molina et al., 2011).

One of the aims of the present study was to better understand the diversity and distribution of BGCs among lichenized fungi, categorize and group PKSs, and identify gene clusters linked to known compounds. We selected three species with overlapping natural product profiles. The upper surface of *P. sulcata, H. physodes*, and *H. tubulosa* is bluish to whitish gray, due to the presence of atranorin in the cortex. The medullary layer contains various colorless depsides and depsidones (Table 1), and the lower surface is black due to the presence of melanins. We hypothesize that some of the BCGs found in the three species are highly similar (orthologous) because they are linked to the same or a structurally similar compound, as has been shown in other, non‐lichenized, fungi (Theobald et al., 2018).

**Table 1:** Lichen substances found in *Parmelia sulcata, Hypogymnia physodes*, and *Hypogymnia tubulosa*. Location in thallus and substance class are given in parentheses.

Lichen species	Natural product	Reference
*Parmelia sulcata*	Atranorin, chloroatranorin (cortical depsides), salazinic acid, consalazinic acid, lobaric acid (medullary depsidones), lecanoric acid (medullary depside)	Brodo et al. (2003), Candan et al. (2007), Duarte (2022), Galloway (2007)
*Hypogymnia physodes*	Atranorin, chloroatranorin (cortical depsides), physodic acid, physodalic acid, 3‐hydroxyphysodic acid, protocetraric acid, fumarprotocetraric acid (medullary depsidones)	Molnár and Farkas (2011), Purvis (1992), Ranković et al. (2014), Solhaug et al. (2009)
*Hypogymnia tubulosa*	Atranorin, chloroatranorin (cortical depsides), physodic acid, 3‐hydroxyphysodic acid, 4‐*O*‐methyl physodic acid (medullary depsidones)	Purvis (1992), Stojanović et al. (2018)

The identified BGCs of *P. sulcata* were compared for orthologs with previously published metagenomes from *H. physodes and H. tubulosa* (Ahmad et al., 2023). Table 1 summarizes the natural products previously reported in the compared lichen. Some of these exhibit medicinally relevant activities (Ranković et al., 2014; Stojanović et al., 2018; Yilmaz et al., 2005). Consequently, this syntenic comparison provides further insights into relevant natural product formation rendering the biosynthetic potential of the three lichen mycobionts from *P. sulcata, H. physodes*, and *H. tubulosa* accessible for biotechnological exploitation.

## 2 MATERIAL AND METHODS

### 2.1 Lichen sample collection

Samples were collected in Germany, Altenschneeberg (August 2022), from the bark of conifers. Precise locations of sequenced samples are latitude 49°26'14.3“N and longitude 12°32'50.9“E. To ensure correct lichen identity, a BLAST search on ITS sequences (see Supporting Information: Table S1: 10.5281/zenodo.8205254) was performed. The lichen sample included in this study was identified as PSU.

### 2.2 GC‐MS analysis of lichen compounds

Furthermore, a part of the collected samples were subjected to GC‐MS analysis to investigate the composition of secondary metabolites. This method was chosen as it enables the identification of volatile compounds in lichen, such as orsellinic acid derivatives. Therefore, approximately 500 mg of dry lichen biomass was macerated in 10 mL of methanol for 24 h at 300 rpm. The resulting extract was then analyzed using a Trace GC‐MS Ultra system with DSQII (Thermo Scientific). An autosampler TriPlus was utilized to inject a sample volume of 1 µL in split mode onto an SGE BPX5 column (30 m, ID 0.25 mm, film 0.25 µm). The injector temperature was set at 280°C. The initial oven temperature was maintained at 50°C for 2.5 min, followed by a temperature increase at a rate of 10°C/min until reaching 320°C, with a final hold step for 3 min. Helium was used as the carrier gas with a flow rate of 0.8 mL/min and a split ratio of 8. Mass spectra and chromatograms were recorded using electron ionization at 70 eV. Masses were detected within the range of 50*m*/*z* to 650*m*/*z* in positive mode (Ringel et al., 2020). Identification of compounds was achieved by comparing their spectra with the NIST/EPA/NIH MS library version 2.0. Identified compounds in Supporting Information: Table S2 depict the top 10 hits found in this sample (Supporting Information: Table S2: 10.5281/zenodo.8205254). Some of these compounds however depict silylated molecules that do not belong to this sample, as no silylation was conducted. These are residuals from previous experiments, which remained on the liner or the column.

### 2.3 High molecular weight DNA (HMW gDNA) extraction and library preparation

Before DNA extraction, the lichen thallus was examined under a binocular microscope to eliminate any moss, wood, and other lichens present in the sample. Additionally, visibly infected parts of the thallus were removed to minimize potential contaminants.

HMW gDNA extraction was conducted as follows. The Quick‐DNA Fungal/Bacterial Miniprep Kit (Zymo Research, Europe GmbH) was used to extract lichen HMW genomic DNA. Dry thallus material from PSU samples was ground into a fine powder using liquid nitrogen. The homogenized material was then transferred to the Bashing Bead Buffer provided in the kit. Genomic HMW DNA was isolated according to the manufacturer's instructions. Due to the high content of polysaccharides, phenolic compounds, and pigments, additional purification steps were necessary. These purifications were performed using the Genomic DNA Clean and Concentrator‐10 Kit (Zymo Research, Europe GmbH) and the DNeasy PowerClean Clean‐up Kit (Qiagen). The quality of the obtained HMW genomic DNAs was assessed using a Nanophotometer (Implen, Nanophotometer Pearl), Qubit 2.0 Fluorometer (Thermo Scientific), and TapeStation (Agilent Technologies).

SMRT bell libraries were constructed for the samples that passed the quality control, which included a 260/280 absorbance ratio of 1.75–1.85 and a 260/230 absorbance ratio of 2.0–2.2. The library was prepared following the instructions for the Low DNA Input Protocol of the SMRT bell Express Prep kit v2 (Pacific Bioscience). Total input DNA with a size range of 10–18 kb for library generation was approximately 350–600 ng. Ligation with T‐overhang SMRT bell adapters was performed at 20°C for 1 h. Following two cleanup steps with AMPure PB beads, the size and concentration of the final library were assessed using TapeStation and Qubit Fluorometer 2.0 with Qubit dsDNA HS reagents Assay Kit.

### 2.4 Genome and RNA Illumina short‐read sequencing

The obtained library was subjected to whole‐genome sequencing on a PacBio Sequel IIe device (Pacific Bioscience). Pre‐extension and adaptive loading (target of p1 + p2 = 0.95) were set to 2 h with an on‐plate concentration of 90 pM. The movie time was set to 30 h (Ritz et al., 2023).

Additionally, RNA sequencing was conducted using short‐read Illumina technology (NovaSeq, Illumina). This transcriptomic data were generated to provide additional depth and accuracy to the predicted gene models. For RNA extraction, frozen lichen thalli were ground using a CryoMill (Retsch), and the RNeasy Plant Mini Kit (Qiagen) was used. To further purify the extracted RNA, the Turbo DNA‐free Kit (Invitrogen) was employed. For short‐read RNA sequencing, the samples underwent processing on a NovaSeq instrument using a paired‐end run mode and a read length of 2 × 150 bp. To begin, total RNAs were extracted using TRI Reagent (Zymo Research, Europe GmbH) following the manufacturer's instructions. Subsequently, the samples underwent further purification using the RNA Clean and Concentrator‐5 Kit (Zymo Research, Europe GmbH). This purification step was repeated until it had a 260/280 absorbance ratio between 1.9 and 2.1, as well as a 260/230 absorbance ratio between 1.8 and 2.2. Only RNAs with a RIN value greater than 8.0 (TapeStation) were considered suitable for sequencing.

### 2.5 Bioinformatic and statistical analysis

Metagenomic reads derived from entire lichen thalli primarily consist of fungal sequences originating from the mycobiont (Greshake Tzovaras et al., 2020). This composition presents challenges for genome assemblers that rely on solid *k*‐mers. The abundance of certain species can lead to their over‐representation, while low‐abundance species like the photobiont may fail to assemble. To address these challenges, the obtained long CCS reads from PacBio Sequel IIe were therefore assembled using metaFlye v2.9.1. This assembler is specifically designed to manage read coverages with high nonuniformity, making it well‐suited for this case. Additionally, the assembled contigs were simultaneously scaffolded using flye, enabling further bioinformatic processing (Kolmogorov et al., 2020).

To differentiate the acquired data sets, a taxonomic binning approach was employed. This involved performing blastx using the DIAMOND v2.0.14 algorithm (Buchfink et al., 2015) on a custom‐made database, as well as utilizing the MEGAN6 LR Community Edition v6.21.7 (Huson et al., 2018). The DIAMOND database used in the analysis encompassed protein sequences from various taxonomic groups, including fungi, bacteria, archaea, viruses, chlorophyta, klebsormidophyceae, tremella, and cystobasidium. To circumvent obstacles like insertion and deletion errors in long‐read sequencing, the flags *‐‐more‐sensitive ‐‐frameshift 15* and *‐‐rage‐culling* were employed in DIAMOND to allow for a frame‐shift‐aware alignment mode (Bağcı et al., 2021). The resulting files were subjected to further processing in MEGAN, where taxonomically assigned sequences were matched to their respective bins (Bağcı et al., 2019). Subsequently, contigs and scaffolds corresponding to the desired nodes were extracted for subsequent analysis. To evaluate the completeness and quality of the resulting bins for further investigation, several tools were utilized, including BUSCO v5.3.2 (Benchmarking Universal Single‐Copy Orthologs) (Simão et al., 2015), QUAST, v5.2.0 (Quality Assessment Tool) (Gurevich et al., 2013), and SeqKit v2.3.1 (Shen et al., 2016). To validate the identities of the mycobiont, an ITSx v1.1.3 analysis was performed (Bengtsson‐Palme et al., 2013). Gene prediction was carried out using AUGUSTUS v3.4.0/BRAKER v2.1.6, leveraging both the metagenomics data and corresponding transcriptomic data as hints (Brůna et al., 2021; Hoff et al., 2016, 2019). This facilitated the functional annotation of genes in the respective data sets. Furthermore, the identification of BGCs present in the obtained bin was performed using antiSMASH v6.1.1 (Blin et al., 2021). The Gene Ontology (GO) terms were levied by InterProScan (v5.59‐91.0) (Jones et al., 2014).

To visualize the distribution of BGCs in all three mycobionts, an evaluation with BiG‐SCAPE (Navarro‐Muñoz et al., 2019) and Cytoscape (Shannon et al., 2003) was conducted, clustering the respective contigs by shared name. To gain insights into the polyketide synthesis in the mycobiont, PKS‐related BGCs were investigated further. At first, a Reciprocal Best Hit (RBH) based on BLAST was performed on the respective primary mycobiont bins of PSU, *Hypogymnia physodes* (HPH), and *Hypogymnia tubulosa* (HTU) (Camacho et al., 2009; Cock et al., 2015) in all three combinations. The latter two were previously published (Ahmad et al., 2023). AntiSMASH, InterProScan, ITSx, SeqKit, and RBH analyses were performed on the Galaxy servers (Afgan et al., 2022). Obtained pairs from RBH were compared for duplicates, highlighting highly conserved PKS‐related genes in the investigated mycobiont genomes. Therefore, the sequences of KS regions were extracted and compared on a phylogenetic level to already published data sets (Gerasimova et al., 2022; Mosunova et al., 2022; Singh et al., 2022) and sequences from the MIBiG database (Terlouw et al., 2023). Therefore, a midpoint rooted maximum likelihood phylogenetic tree (IQ‐TREE v2.1.2; Nguyen et al., 2015), levied with 1000 ultrafast bootstraps (Minh et al., 2013) and the elaborated substitution model (ModelFinder; Kalyaanamoorthy et al., 2017) LG + F + G8 was built based on an alignment with MAFFT v7.508 (Katoh & Standley, 2013). The included sequences were grouped in the vicinity of the KS sequences from HPH, HTU, and PSU to highlight the validity of the obtained results. It yielded the following metrics, based on Input data of 76 sequences with 3742 amino‐acid sites, exhibiting 917 constant and invariant sites, 2142 parsimony informative sites, and 3009 distinct site patterns. This tree was visualized with iTOL v5 (Letunic & Bork, 2021) and Inkscape. The according fasta and alignment file is provided in Supporting Information: Tables S3 and S4: 10.5281/zenodo.8205254. Subsequently, an alignment of related gene clusters was performed on EasyFig (v2.2.5) (Sullivan et al., 2011) to investigate PKS synteny between HPH, HTU, and PSU. Additionally, a progressiveMAUVE (Darling et al., 2004) alignment of the whole mycobiont bins was deployed to visualize synteny on the genome level in Geneious (Geneious Prime® 2022.0.1).

## 3 RESULTS AND DISCUSSION

### 3.1 Genome sequencing and quality assessment

The same lichen‐related substances were observed in the GC‐MS analysis (see Supporting Information: Figure S1 and Table S2: 10.5281/zenodo.8205254) of PSU as in the previously described species HPH and HTU (Ahmad et al., 2023). Therefore, this lichen was deemed to harbor familiar BGCs. To present sequencing quality, the metrics of PSU are listed in Table 2. As the mean HiFi Read Quality exhibited a value well above Q20, further bioinformatic analyses were conducted.

**Table 2:** Metagenomic PacBio sequencing of *Parmelia sulcata* (PSU).

Analysis metrics	PSU
Total bases (Gb)	683.00
HiFi reads	3,799,596
HiFi yield (Gb)	26.01
HiFi read length (mean, bp)	6845
HiFi read quality (median)	Q43
HiFi number of passes (mean)	18

After taxonomic analysis, the mycobiont (Parmeliaceae) bin was further investigated. Evaluating the contiguity of the assembled metagenome involved the utilization of the quality assessment tool for genome assemblies (QUAST). The metagenome was assessed based on the genome size, number of contigs, and N50 values. A summary of the statistics is provided in the upper part of Table 3. Notably, the N50 value was beneath the recommended threshold of 1 Mb from PacBio, indicating poor contiguity. To address this issue, ITS analysis was performed yielding two ITS sequences, which were both identified as PSU in the NCBI BLAST search against the nr database using megablast. This may be due to the intertwined growth of two closely related subspecies of PSU or the lack of data to align the respective ITS sequences to. Subsequent binning clustered all the related contigs together, which may have impacted the QUAST results regarding, for example, N50. Nevertheless, the low L50 value suggests sufficient contiguity of the obtained genome. The possible presence of two closely related lichen species in the mycobiont bin is further supported by a comparison of the obtained genome size to that of *Parmelia* spp. reported in the literature (45 Mb, NCBI BioSample: SAMN17391792). Average coverage of 107× was deemed to be highly sufficient for further processing. Comparison of GC content with the genome from literature yields equivalent results. For further assessment, a gene prediction was conducted with Augustus‐BRAKER based on short‐read RNA sequencing data. The resulting statistics are depicted in the lower part of Table 3. The observed gene count was 24,437, which exceeded the median value of 11,000 (Stajich, 2017), further hinting at the presence of two mycobionts in the investigated bin.

**Table 3:** QUAST and gene prediction results of Parmeliaceae bin, including the mycobiont sequences.

	Analysis metrics	Parmeliaceae
QUAST	Number of contigs	328
	Largest contig	2,191,686
	Total length	82,929,871
	N50	698,771
	L50	41
	Number of Ns per 100 kbp	0
	Average coverage	107.9
	GC content	47.62
Gene prediction	Gene count	24,437
	Average gene length	1547.1
	Gene density (genes/Mb)	294.67
	Introns/gene	2.29

**Figure 1:**
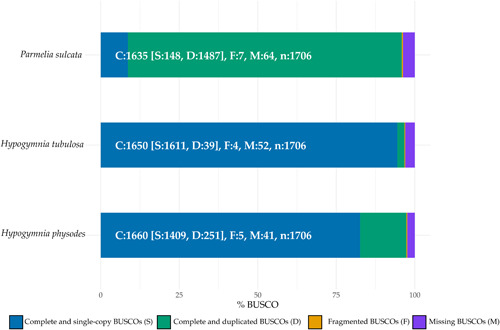
BUSCO genome completeness assessment of the mycobiont subsets of *Parmelia sulcata, Hypogymnia tubulosa*, and *Hypogymnia physodes*; the latter two were previously described (Ahmad et al., 2023). The subset checked for completeness was Ascomycota with the orthologous gene set ascomycota_odb10.

Genome completeness and reliability for further data processing were evaluated with a BUSCO (Simão et al., 2015) assessment. The results were normalized and summarized in Figure 1. Ascomycota odb10 was utilized as the orthologous gene set. Obtained BUSCO results are depicted in percentages to allow for an accurate comparison between the investigated bins. Additionally, absolute numbers were included in all columns to allow for more precise comparison between different bins.

At first sight, PSU exhibits a high number of complete and duplicated BUSCOs supporting the statement of two closely related PSU species. The remaining bins harbored more complete and single BUSCOs. The fragmentation rate was low throughout all investigated mycobionts. The sum of missing BUSCOs was comparable in the three bins.

### 3.2 BGC  annotation and phylogeny

**Figure 2:**
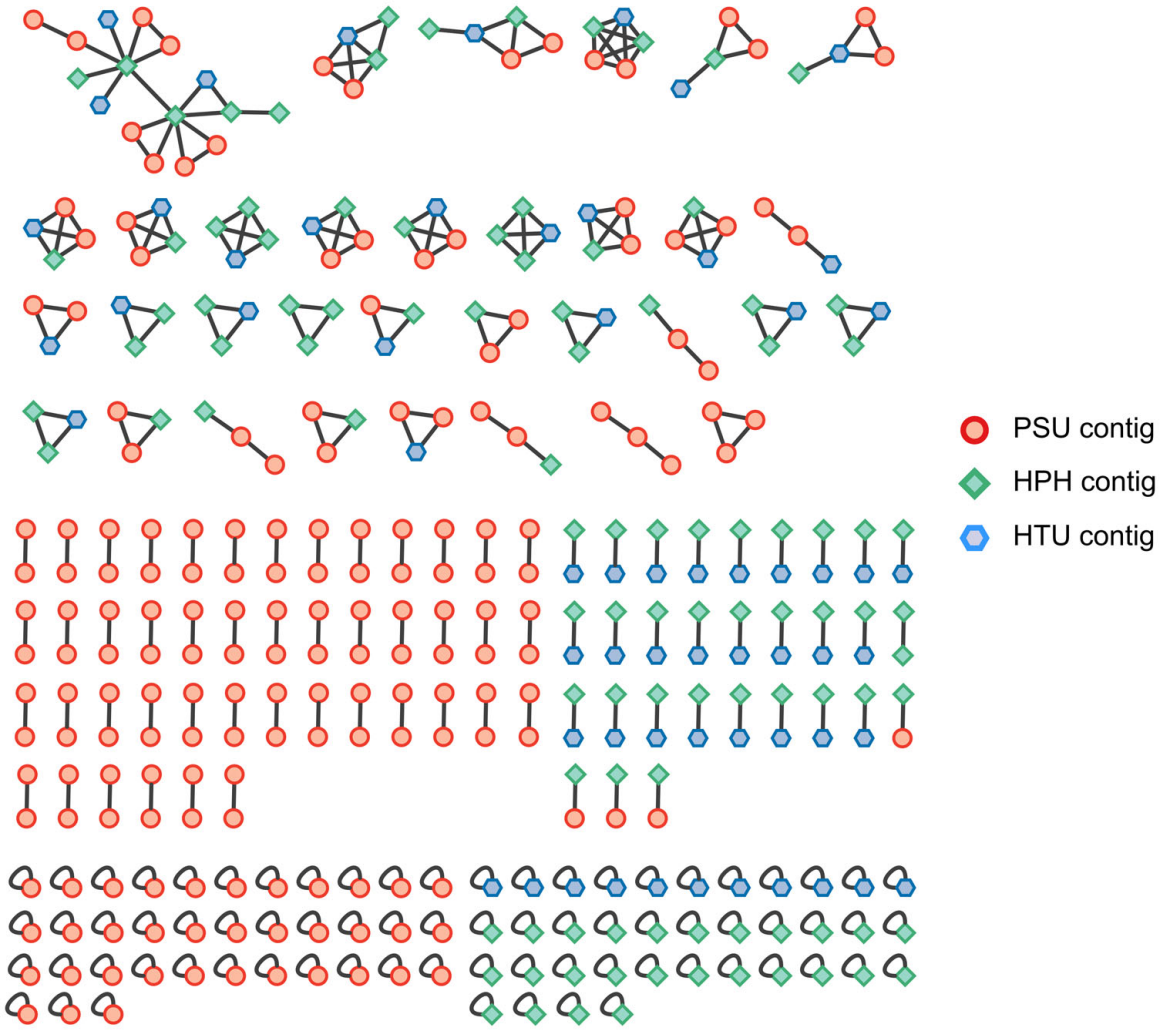
BiG‐SCAPE analysis and visualization of biosynthetic gene clusters (BGCs). Here, BGCs of all three lichen mycobionts were analyzed in BiG‐SCAPE and clustered in Cytoscape. The upper segment represents the similarity network between the contigs of the investigated three lichen mycobionts. In the left middle segment, contig pairs of *Parmelia sulcata* (PSU) are grouped, whereas on the right side, the pairs of *Hypogymnia physodes* and PSU are located. The singletons of each mycobiont are depicted in the lower section. These represent contigs with no significant sequence similarity to others in the pool.

The genus *Parmelia* produces a plethora of secondary metabolites, rendering further evaluation of inherent pathways highly interesting (Candan et al., 2007; Elečko et al., 2022; Gandhi et al., 2022; Ranković & Kosanić, 2019; Ranković et al., 2007). Therefore, the obtained metagenome of *P. sulcata* was investigated for BGCs by antiSMASH 6.1.1 fungal/bacterial version (Blin et al., 2021), leveraging the prior conducted gene annotation. This yielded the following amount of BGCs for the three mycobiont bins: HTU 73, HPH 114, and PSU 214. These findings are coherent with the observation of enhanced secondary metabolite production in lichen mycobionts and fungi (Bills & Gloer, 2017; Devi et al., 2020; Goga et al., 2020; Ola et al., 2013; Shwab & Keller, 2008). The number of BGCs present in HPH and HTU from previous studies (Ahmad et al., 2023) was in line with the literature, as the described BGC content in lichen ranges between 27 and 80 (Calchera et al., 2019). Regarding PSU, the total number of BGCs exceeded others potentially due to the bin containing two closely related mycobionts. As *P. sulcata* is a widely distributed lichen species and known for its high variability, the possibility of collecting two closely related, intricately growing species is high (Brodo et al., 2003). These are referred to as “cryptic” and are described as different species with similar morphology (Bickford et al., 2007; Brodo et al., 2003; Hawksworth & Rossman, 2007; Molina et al., 2011). The phenomenon of cryptic species is often observed in *P. sulcata* (Molina et al., 2011). This renders the physical isolation of only one specimen from an environmental sample challenging or even not feasible. The same applies to separation on a metagenomic level, as most currently available genomes were assembled de novo and the overall amount is still limited, making binning on a species level challenging. However, if the amount of BGCs was divided by two, more comparable results may be obtained. By comparison to different organisms, which also exhibit a high richness in BGCs like *Nocardia* spp. (~36) (Doroghazi & Metcalf, 2013; Männle et al., 2020), *Myxobacteria* spp. (30–46) (Amiri Moghaddam et al., 2018; Gregory et al., 2019), *Streptomyces* spp. (23–80) (Caicedo‐Montoya et al., 2021; Liu et al., 2021), and *Cyanobacteria* spp. (1–42) (Dittmann et al., 2015; Popin et al., 2021), the presented mycobiont genome shows comparable or higher totals of BGCs when the nature of the deployed bin is considered. For the new genome of PSU, antiSMASH assigned 10 out of 214 BGCs with a specific function (100% similarity to MIBiG clusters), leaving the majority of the BGCs with unknown or uncharacterized functions. Among the annotated “most similar cluster” were, for example, 6‐hydroxymellein,1,3,6,8‐tetrahydroxynaphthalene, and clavaric acid. Please refer to Supporting Information: Table S5 for further information: 10.5281/zenodo.8205254. A visual representation of all BGCs from the three investigated mycobionts is depicted in Figure 2. The analysis with BiG‐SCAPE yielded a similarity network which was visualized with Cytoscape. Investigated BGCs were color‐coded based on their respective mycobiont. This visualization highlights the connection of various BGCs between the investigated mycobionts. It comes to attention that only one cluster contains solely one BGC belonging to PSU; all others contain two BGCs from PSU if this mycobiont is involved. By focusing on the middle segment of Figure 2, it becomes evident that PSU exhibits a high amount of paired BGCs (45) and also triples (two, located at the lower part of the upper segment), which is not observed in the other two mycobionts. Due to the high duplicates but also the presence of singletons grouped in the lower part of the figure, the assumption on two closely related *P. sulcata* species is fortified, as singletons exhibit no sufficient sequence similarity to other BGCs present in the investigated data set. These unique or highly divergent BGCs do not share common biosynthetic gene homology with other clusters in the data set. This renders them highly interesting for further investigation as these may provide insights into the potential production of novel or uncharacterized secondary metabolites (Sánchez‐Navarro et al., 2022). Since the most abundant group of BGCs is related to polyketide formation, these were further investigated.

Subsequently, BGCs related to PKS formation were subjected to RBH with BLAST (Camacho et al., 2009), where all three mycobiont data sets were compared and paired. The resulting orthologous pairs yielded highly conserved regions accessible for further analysis (Supporting Information: Table S6: 10.5281/zenodo.8205254).

**Figure 3:**
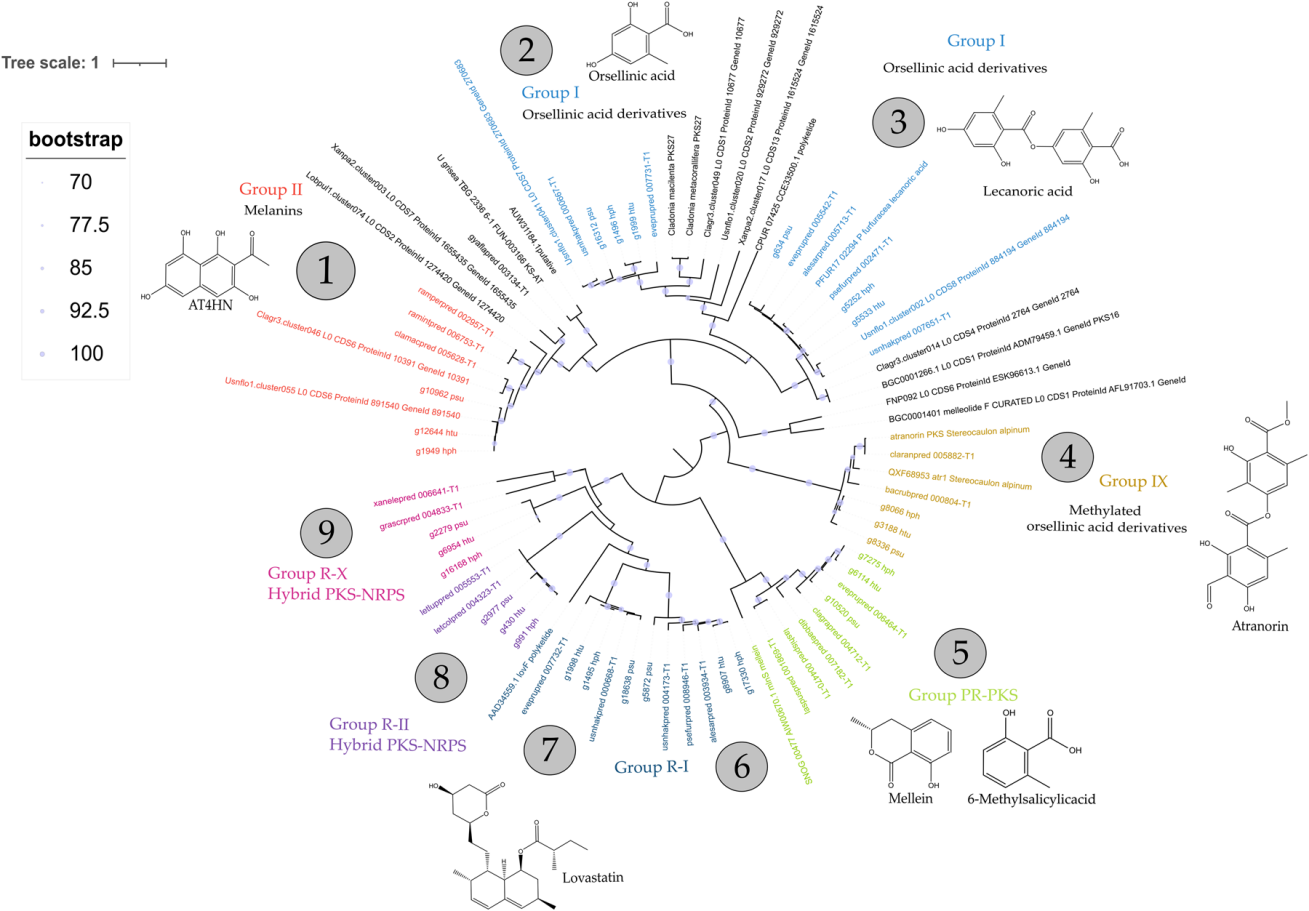
Phylogenetic tree of orthologous KS regions compared to reference sequences. Phylogenetic relationships of nine biosynthetic gene clusters (Clades 1–9), which had an ortholog in each of the three studied species. The phylogeny is based on the ketosynthase domain of the PKS. Gene names were tagged with the abbreviation of the respective lichen mycobiont. Obtained KS sequences were compared on a phylogenetic level with the data sets from previous studies, including sequences from the MIBiG database, and the groups were named accordingly (Gerasimova et al., 2022; Mosunova et al., 2022; Singh et al., 2022). All clades were allocated to the respective group with the corresponding putatively produced chemical compound. AT4HN, 2‐acetyl‐1,3,6,8‐tetrahydroxynaphthalene.

As the ketosynthase (KS) region of PKS represents their most conserved domain (Amnuaykanjanasin et al., 2009), a phylogenetic tree was constructed based on these sequences (Figure 3). Depicted clades were numbered from one to nine and additionally included gene names tagged with the lichen mycobiont they are derived from. In every clade, three KS regions belonging to the different mycobionts aligned with reference sequences. These findings strongly highlight the degree of similarity between the compared PKS throughout all examined samples. In addition, both *Hypogymnia* species clade more closely than the PSU sample, which reflects the phylogenetic relationships (Divakar et al., 2015). Furthermore, elaborated KS regions were compared phylogenetically to previous studies to elucidate putative function and natural product formation (Gerasimova et al., 2022; Mosunova et al., 2022; Singh et al., 2022). This tree depicts the KS regions of the lichen compared in this study with reference sequences from previous studies (Gerasimova et al., 2022; Mosunova et al., 2022; Singh et al., 2022) and the MIBiG database. The upper part of Figure 3 depicts PKS belonging to the group of nonreducing PKS (NR‐PKS), comprising Clades 1–4. The other part of the figure shows Clades 6–9 representing reducing PKS (R‐PKS), whereas Clade 5 contains a partially reducing PKS (PR‐PKS). The position of the latter is in line with the nature of the described reductive functions, being in the middle of the two types. Clade names are based on previous PKS phylogenies (Gerasimova et al., 2022; Mosunova et al., 2022; Singh et al., 2022). Clade 1 is putatively linked to naphthalene‐like compounds, which is also in line with annotations by antiSMASH. This BGC may be associated with melanin biosynthesis in the three studied lichenized fungi. All of the species are characterized by a black (melanized) lower surface of the thallus (Elvebakk, 2011). Clades 2 and 3 belong to Group I and are linked to orsellinic acid and its derivatives, such as the di‐depside lecanoric acid (Schroeckh et al., 2009) and the tri‐depside gyrophoric acid (Singh et al., 2022). We observed orsellinic acid in the GC‐MS analysis. Orcinol‐type depsidones may also be linked to these clusters (Singh et al., 2021a). Interestingly, the BGCs in Clade 2 contain a second, reducing PKS (Figure 3). Alternatively, a FAS comprising HexA and HexB subunits could be responsible for the synthesis of the acyl chains, as seen in the biosynthesis of norsolorinic acid in the aflatoxin pathway (Brown et al., 1996; Watanabe & Townsend, 2002). Homologs of HexA and HexB genes have been found in the lichenized fungi *Pseudevernia furfuracea* and *Cladonia grayi* (Singh et al., 2021a). Clade 4 contains a PKS with a cMet domain and is linked to methylated orsellinic acid and its derivatives, for example, the beta orcinol‐type depside atranorin. This BGC has been functionally characterized and was found in the genomes of several atranorin‐producing lichens (Kim et al., 2021). Regarding Clade 5 (PR‐PKS), a possible product is mellein or 6‐methyl salicylic acid (6‐MSA), resembling other polyketides produced by lichen. For the R‐PKS, Clades 6, 7, and 8 were assigned to R‐I and R‐II, allegedly producing lovastatin‐like compounds. Interestingly, Clade 9 was putatively assigned to the novel R‐PKS group X being located at the very edge of this group, estimating product formation challenging. It is to be mentioned that biosynthetic core genes of Clades 8 and 9 were annotated as hybrid PKS‐NRPS. To further validate the obtained results, the whole PKS sequences of HPH, HTU, and PSU allocated to the respective clades were compared with some of the reference sequences grouping in the vicinity of these via blastp and another phylogenetic tree (Supporting Information: Figure S4: 10.5281/zenodo.8205254). The phylogenetic tree only slightly differs from Figure 3 in the position of Clade 8 and the similarity of Clade 8 to Clades 6 and 7. Deviations in the tree are due to differences in sequence homology resulting in a light disposition of the clades. The blast results (Supporting Information: Table S7: 10.5281/zenodo.8205254) yielded percent identities of an average of 65%, excluding alignments with the lovastatin PKS lovF in Clades 6 to 8 (average 32%) and the reference sequences in Clade 9 (average 40%). This also explains the position of the identified genes of HPH, HTU, and PSU in Clade 9 in relation to the reference sequences. With the nevertheless high homology in KS domains, an investigation on the synteny level may permit a deeper insight into the homology of PKS‐related BGCs.

### 3.3 Synteny of investigated mycobiont BGCs

As the distribution of presented BGCs in Figure 2 slightly differs, an investigation of synteny on the genomic level may yield more insights into conserved regions throughout the evaluated mycobionts. In Supporting Information: Figures S2 and S3 (10.5281/zenodo.8205254), the bin alignments of HPH/HTU and HTU/PSU with progressiveMAUVE (Darling et al., 2004) were pictured. The red bars in the alignments indicate regions of large‐scale rearrangements or inversions, which were predominantly observed between HPH and HTU. By comparing the number of lines connecting the synteny blocks of the respective lichen mycobiont pair, the close relation of HPH and HTU becomes more prominent while a lower amount connects the blocks of HTU and PSU. As HPH and HTU share a high synteny, only a comparison of HTU and PSU was attached as an additional comparison with HPH was considered redundant.

**Figure 4:**
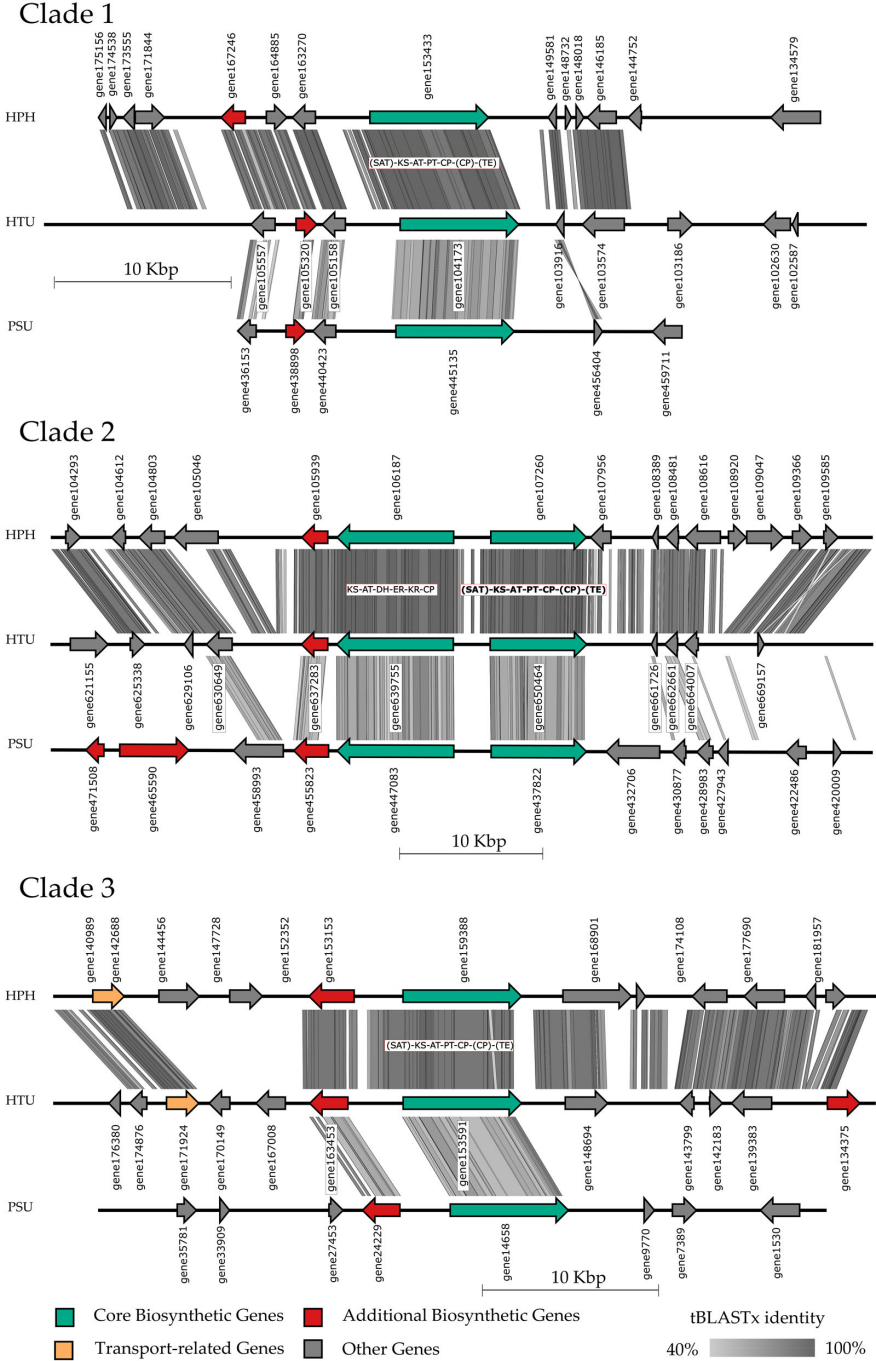
Synteny plot of Clades 1–3 *of* the orthologous PKS genes. Three out of nine highly syntenic biosynthetic gene clusters found in the three studied lichen‐forming fungi. Clade 1 is linked to melanin biosynthesis, and Clades 2 and 3 are linked to the synthesis of orsellinic acid and related orcinol‐type depsides and depsidones. Phylogenetic relationships of the nine clusters (based on the ketosynthase domain of the T1PKS core gene) *and putatively* associated compounds are presented in Figure 3. ACP, acyl carrier protein; AT, acyltransferase; CP, carrier protein; DH, dehydratase; ER, enoylreductase; KR, ketoreductase; KS, ketosynthase; PT, product template; SAT, starter unit:acyl‐carrier protein transferases; TE, thioesterase. Domains in brackets were annotated via antiSMASH as outside or incomplete modules.

To gain deeper insights into the conserved PKS of the investigated mycobionts, a synteny evaluation of the BGCs derived from the phylogenetic tree (Figure 3) was conducted. The resulting synteny plots were constructed using EasyFig and comprise each of three of the phylogenetic clades. All plots include gene names with correlating sequences, which can be found in the Supporting Information of the respective mycobiont (please refer to Supporting Information: Table S8 and Folder S1: 10.5281/zenodo.8205254). Additionally, the domain composition of the homologous core gene was included, highlighting the phylogenetically allocated core gene in bold font for BGCs with more core genes. A high cluster homology can be observed between HPH and HTU throughout all computed synteny plots. In Figure 4, the core biosynthetic genes are conserved in all three clades, which also applies to additional biosynthetic genes. The cluster homology between HTU and PSU is mostly confined to the annotated genes. However, several HPH coding regions in Clade 1, for example, exhibit high homology to noncoding regions in HTU. These may be artifacts from gene prediction, indicating genes missed by annotation (Calchera et al., 2019). For Clade 1 and Clade 2, an inversion is observed while comparing HTU to PSU. The domain composition of orthologous core genes in Clade 1, Clade 3, and the first core gene (bold) of Clade 2 indicates that the PKS is nonreducing. On the other hand, the remaining Clade 2 core gene suggests reducing the activity of the corresponding PKS.

**Figure 5:**
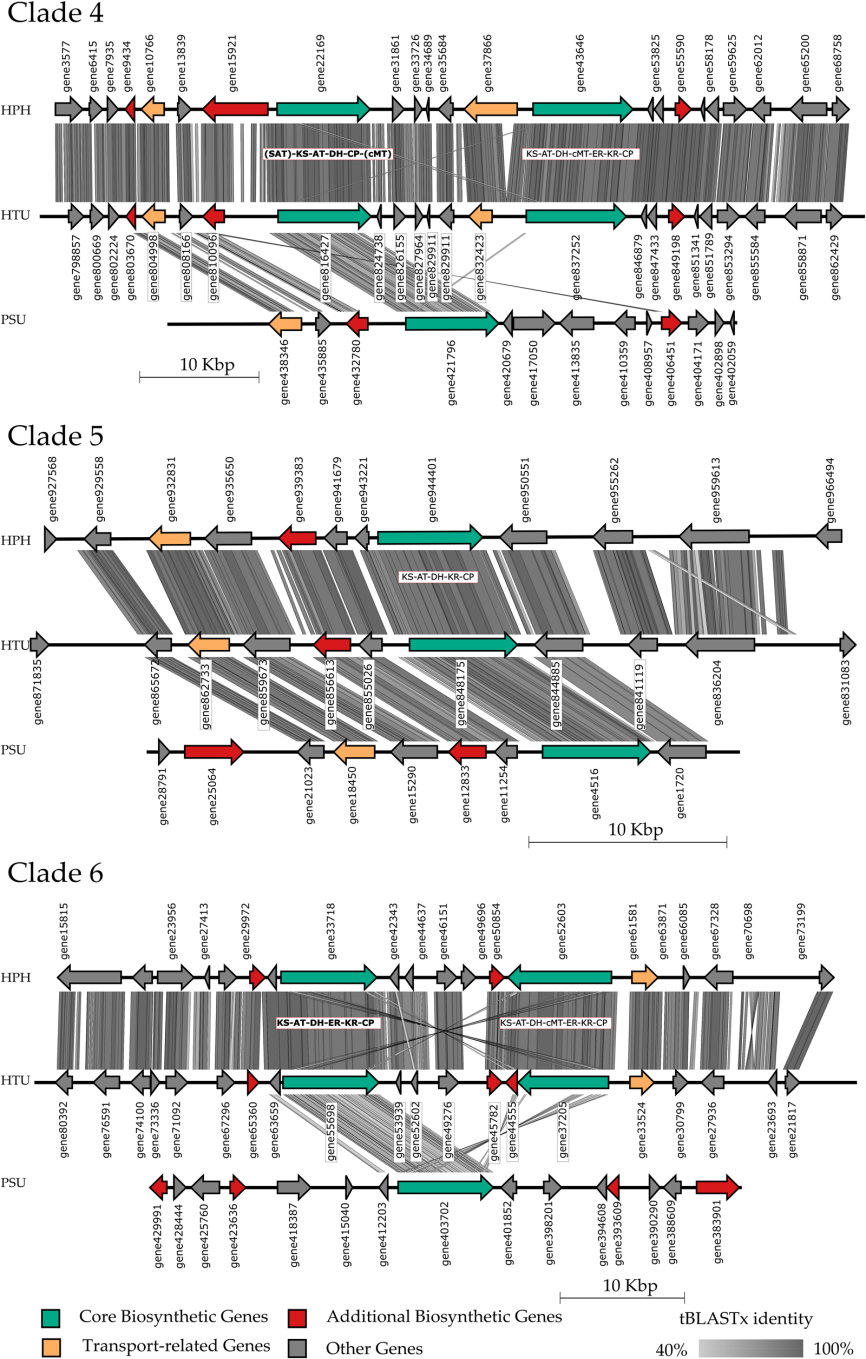
Synteny plot of clades 4–6 of the orthologous PKS genes. Three out of nine highly syntenic biosynthetic gene clusters found in the three studied lichen‐forming fungi. Clade 4 is linked to the synthesis of methylated *orsellinic acid* and related beta‐orcinol‐type depsides, such as atranorin. Clade 5 is linked to mullein or 6‐MSA biosynthesis, whereas Clade 6 putatively produces lovastatin‐like compounds. Phylogenetic relationships of the nine clusters (based on the ketosynthase domain of the T1PKS core gene) and putatively associated compounds are presented in Figure 3. AT, acyltransferase; cMT, C‐methyltransferase; CP, carrier protein; DH, dehydratase; ER, enoylreductase; KR, ketoreductase; KS, ketosynthase; SAT, starter unit: acyl‐carrier protein transferases. Domains in brackets were annotated via antiSMASH as outside or incomplete modules.

Regarding cluster homology in Figure 5, similar results are observed as in Figure 4, with HPH and HTU depicting high sequence homology. PSU exhibits the highest cluster homology in Clade 5 when compared to the remaining two clades. In addition to the core genes, accessory genes show high homology to HPH and HTU. The PKSs in the investigated clades represent partially reducing activities in Clade 5 and reducing PKS in Clade 6, whereas Clade 4 exhibits a nonreducing PKS based on the sequence of domains. The distribution and composition of genes (core and accessory) differ between PSU and the remaining mycobionts, in particular Clades 4 and 6.

**Figure 6:**
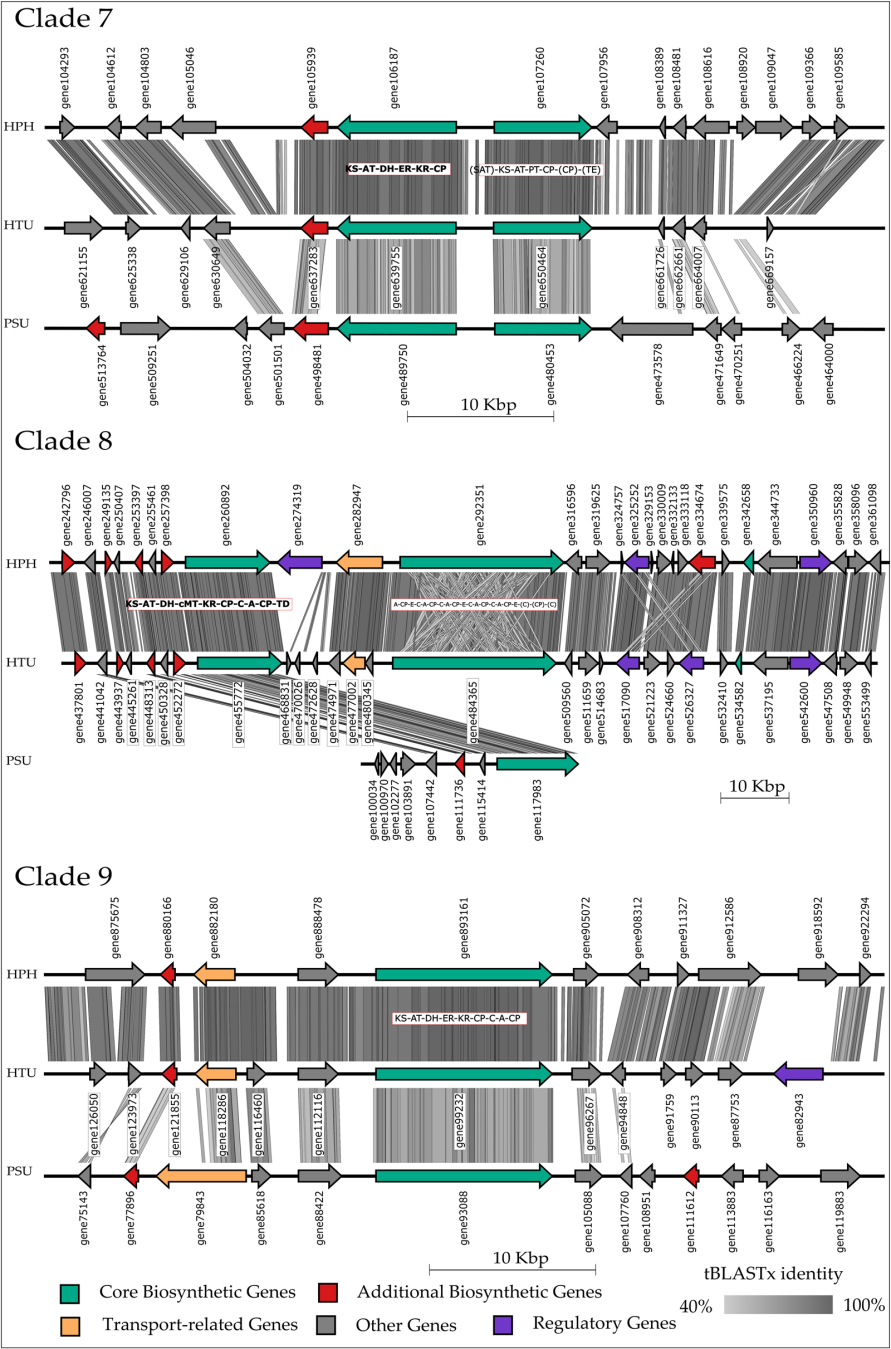
Synteny plot of clades 7–9 of the orthologous PKS genes. Three out of nine highly syntenic biosynthetic gene clusters found in the three studied lichen‐forming fungi. Clades 7 and 8 are linked to the putative synthesis of lovastatin‐like compounds. Clades 8 and 9 are annotated as hybrid PKS‐NRPS BGCs. A, AMP‐binding; AT, acyltransferase; C, condensation; cMT, C‐methyltransferase; CP, carrier protein; DH,  dehydratase; ER, enoylreductase; KR, ketoreductase; KS, ketosynthase; TD,  terminal domain. Domains in brackets were annotated via antiSMASH as outside or incomplete modules.

The last group of clades is visualized in Figure 6. Notably, Clades 2 and 7 include the same genes from HPH and HTU but different genes from PSU. Homology between HPH and HTU is comparable to the previously described figures while PSU differs. The BGC of PSU in Clade 8 is small when compared to those of HPH and HTU; however, the orthologous core genes express high homologies, including some accessory genes. Clade 9 exhibits high similarities in gene composition throughout all compared. In Clades 8 and 9, only regulatory genes were annotated throughout all investigated clades. The absence of regulatory genes in the other BGCs may have several reasons. Possibly these genes were incompletely annotated or artifacts in gene prediction persisted. Other potential causes involve a distributed regulation from external regulatory genes (Sun et al., 2022) or an alternative, non‐gene‐based regulatory mechanism (Wang et al., 2021). Another conceivable reason may be a novel or yet uncharacterized regulatory gene (Keller, 2019).

By combining phylogenetic analyses with subsequent synteny evaluation of the KS regions, we were able to demonstrate that gene cluster similarity strongly correlates with the underlying KS topology (Calchera et al., 2019; Jenke‐Kodama et al., 2005; Kroken et al., 2003; Ziemert & Jensen, 2012). Compared BGCs pose an intriguing opportunity for further investigation of polyketide synthesis. As these derive from lichen with comparable secondary metabolite production according to GC‐MS, the underlying genes for these natural products should be highly conserved, rendering them accessible for experimental exploitation.

## 4 CONCLUSIONS

In this study, we provide a new genome of PSU, which was extracted from a metagenomic sample. Additionally, inherent PKS genes were compared with sequences from *Hypogymnia physodes* and *Hypogymnia tubulosa* from a previous study (Ahmad et al., 2023). Thus, orthologous PKS genes were evaluated on a phylogenetic level with reference sequences from Gerasimova et al., 2022; Mosunova et al., 2022; Singh et al., 2022; and the MIBiG database. The obtained phylogenetic tree provides information about the allocated putatively produced compounds of each clade. While most of the investigated clades show high sequence homology when compared to the respective reference sequences (Gerasimova et al., 2022; Mosunova et al., 2022; Singh et al., 2022), Clades 8 and 9 exhibit low homologies and need further investigation. A syntenic evaluation of the nine orthologous BGC triplets from the three lichen samples compared in this study highlights the similarity of the biosynthetic core genes and renders them ready for wet lab experiments. This genome mining approach identified the sequences involved in the putative formation of various polyketides, which need to be further investigated by expression in suitable organisms in wet‐lab experiments. As lichen secondary metabolites are still yet an untapped pool of compounds with pharmaceutical relevance, this study gives access to a new high‐quality genome ready for genome mining.

Based on these findings, further experiments can be conducted to shed light on the biosynthetic machinery of lichen PKS and their intriguing product spectra. The obtained results can be used to verify the predicted function and help to dereplicate PKS for future studies.

## AUTHOR CONTRIBUTIONS

**Nadim Ahmad**: Conceptualization (equal); data curation (lead); formal analysis (equal); investigation (equal); methodology (equal); software (equal); validation (equal); visualization (equal); writing—original draft (lead); writing—review & editing (equal). **Manfred Ritz**: Conceptualization (equal); formal analysis (equal); investigation (equal); methodology (equal); software (equal); validation (equal); visualization (equal); writing—review & editing (equal). **Anjuli Calchera**: Investigation (equal); methodology (equal); validation (equal); writing—review & editing (equal). **Jürgen Otte**: Investigation (equal). **Imke Schmitt**: Conceptualization (equal); writing—review & editing (equal). **Thomas Brueck**: Funding acquisition (lead); resources (lead); supervision (equal); writing—review & editing (equal). **Norbert Mehlmer**: Conceptualization (equal); project administration (lead); supervision (equal); writing—review & editing (equal).

## CONFLICT OF INTEREST STATEMENT

None declared.

## ETHICS STATEMENT

None required.

## Data Availability

The sequence data presented in this study are openly available in the National Center for Biotechnology Information (NCBI) under BioSample accession numbers: *Parmelia sulcata* SAMN35345252, *Hypogymnia physodes* SAMN34074577, and *Hypogymnia tubulosa* SAMN34074619. Supporting Information: Figures S1–S4 and Tables S1–S8 are available in the Zenodo repository: 10.5281/zenodo.8205254
